# Enzymatic processing of lignocellulosic biomass: principles, recent advances and perspectives

**DOI:** 10.1007/s10295-020-02301-8

**Published:** 2020-08-25

**Authors:** Heidi Østby, Line Degn Hansen, Svein J. Horn, Vincent G. H. Eijsink, Anikó Várnai

**Affiliations:** Faculty of Chemistry, Biotechnology and Food Science, Norwegian University of Life Sciences (NMBU), P.O. Box 5003, 1432 Aas, Norway

**Keywords:** Lignocellulose, Saccharification, Cellulase, Hemicellulose, Lytic polysaccharide monooxygenase

## Abstract

Efficient saccharification of lignocellulosic biomass requires concerted development of a pretreatment method, an enzyme cocktail and an enzymatic process, all of which are adapted to the feedstock. Recent years have shown great progress in most aspects of the overall process. In particular, increased insights into the contributions of a wide variety of cellulolytic and hemicellulolytic enzymes have improved the enzymatic processing step and brought down costs. Here, we review major pretreatment technologies and different enzyme process setups and present an in-depth discussion of the various enzyme types that are currently in use. We pay ample attention to the role of the recently discovered lytic polysaccharide monooxygenases (LPMOs), which have led to renewed interest in the role of redox enzyme systems in lignocellulose processing. Better understanding of the interplay between the various enzyme types, as they may occur in a commercial enzyme cocktail, is likely key to further process improvements.

## Introduction

Industrial-scale production of cellulosic ethanol based on enzymatic saccharification of biomass was established by several companies during the past decade [17, 298]. This production of cellulosic ethanol was initiated in 2012 by Beta Renewables at their site in Crescentino, Italy [55]. In 2015, this plant had an annual production of about 40,000 tons of ethanol using agricultural residues as feedstock. In 2017, however, this plant was shut down due to economic problems in the parent company Mossi Ghisolfi Group and sold to Versalis [107]. In early 2020, Eni, an integrated energy company owning Versalis, announced that bioethanol production in Crescentino will start again within the first half of 2020 [98]. Other companies like DuPont, Abengoa and GranBio have all had commercial plants in operation, but they have closed down production of ethanol due to economic and/or technical reasons. The POET-DSM Advanced Biofuels, a 50/50 joint venture between Royal DSM (Netherlands) and POET LLC (USA) demonstrated stable industrial production of bioethanol. Their Project Liberty facility in Emmetsburg, Iowa (USA) produced for some time around 80 million liters of ethanol per year and had an 80% uptime in 2017. However, also POET-DSM has now paused ethanol production at the site due to challenges with implementing the recent Renewable Fuel Standard [277]. Thus, the establishment of this industry has clearly been challenging, and it is currently also struggling with a low oil price.

**Fig. 1:**
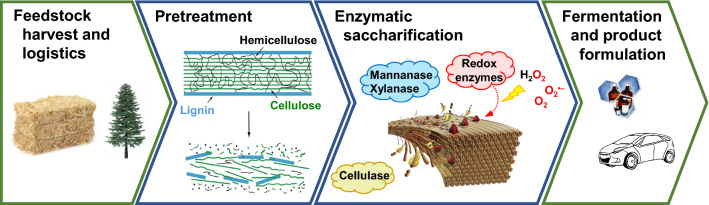
The main steps of the conversion of lignocellulosic biomass to ethanol. Depending on the choice of microorganism in the fermentation step, a range of different fuels and chemicals may be produced

Conversion of lignocellulosic biomass to ethanol involves five main steps, namely collection and delivery of feedstock to the plant, pretreatment of the feedstock (at the point of collection or on-site), enzymatic saccharification, fermentation and product formulation (see Fig. 1). In order to make the process viable, all these steps need to be considered from the economic point of view, with primary focus on feedstock handling, pretreatment and enzyme efficiency and enzyme costs [4, 383]. In this review, we will give an overview of recent technical improvements regarding pretreatment technologies that have been used at (semi-)industrial scale and then discuss in detail challenges and recent advancements regarding enzyme cocktails used for saccharification of lignocellulosic biomass. We will focus on enzyme components that are critical for maximizing sugar recovery from the pretreated feedstock and on the interactions between these components in enzyme mixtures. Finally, we will address the limitations of today’s cellulase cocktails and discuss possible strategies for their improvement.

## Pretreatment technologies and their effect on the feedstock

A broad range of pretreatment technologies is available to enhance accessibility of lignocellulosic biomass to enzymes and hence promote saccharification, as reviewed by Yang and Wyman [389], Sun et al. [328] and Cantero et al. [50]. Among these, wet oxidation [307], hydrothermal pretreatment [270], steam explosion [44, 275], dilute acid treatment [252], ammonia fiber expansion (AFEX) [16], sulfite pulping [301, 377] and methods based on the use of ionic liquids and organic solvents [398] are the major technologies that have been used at demonstration or industrial scale over the past years. The choice of pretreatment depends on the type of feedstock as well as on the spectrum of desired end products [95, 301]. Hydrothermal pretreatment as well as AFEX and ammonium recycle percolation (ARP) technologies cause cellulose decrystallization, some hydrolysis of hemicellulose as well as lignin removal [18] and are primarily used for grass-type biomass (corn stover, switch grass), while steam explosion and alkaline and sulfite pulping can also be used for woody biomass (e.g., poplar and spruce). Recent improvements aim at reducing saccharification costs and include the following: (1) combined removal of lignin and hemicellulose prior to mechanical refining [54, 193, 388]; (2) restructuring native cellulose to the more accessible allomorph cellulose III in a low moisture extractive ammonia (AE) process [78]; and (3) the use of biomass-derived solvents for biomass pretreatment [179, 223, 322]. As an example, a pretreatment process recently developed at NREL [193], which uses a counter-current alkaline deacetylation [194] followed by mechanical defibrillation of the feedstock, allows enzymatic saccharification at high consistency, and the resulting hydrolysate is highly fermentable.

While some pretreatment technologies aim to increase plant cell wall accessibility via reorganization of plant cell wall polymers without removal of matrix polymers (AFEX, ARP), other technologies increase enzymatic accessibility of cellulose via fractionation of the biomass by separating lignin (e.g., alkali and sulfite pulping), hemicellulose (steam explosion) or both (ionic liquid or organosolv pretreatment) from cellulose. Detailed analysis of pretreated biomass with glycome profiling and immunolabeling of plant cell wall polymers indicate that not even the most efficient pretreatment technologies, such as hydrothermal pretreatment [86, 397], AFEX [264] and extractive ammonia pretreatment [13], can completely separate cellulose from the other cell wall polymers. Indeed, studies on the optimization of enzymatic biomass saccharification have revealed the need for a wide-spectrum enzyme cocktail, including cellulases and hemicellulases, to achieve complete saccharification of pretreated biomass, and the composition of the optimal enzyme cocktail depends on pretreatment and biomass type [21, 61, 168].

## The active components of cellulase cocktails

### Cellulolytic enzymes

**Fig. 2:**
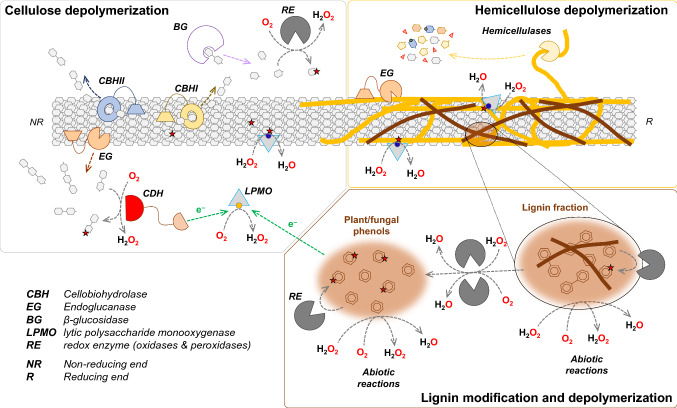
Schematic view of a cellulose fibril covered with hemicellulose (orange) and lignin (brown) and key enzymes involved in the depolymerization of plant cell wall polysaccharides. The non-reducing (NR) and reducing (R) ends of the cellulose chains are marked. Stars indicate oxidation catalyzed by LPMOs (triangles) or other redox enzymes (RE, grey). Orange spheres depict Cu(II) and blue spheres depict Cu(I) in the active site of LPMOs. Interactions between hydrolytic and redox enzymes are indicated. For simplicity, the multitude of hemicellulose-active enzymes, including, e.g., debranching enzymes, are indicated as “hemicellulases”, while lignin-active enzymes are referred to as redox enzymes (“RE”). Note that fungal secretomes may contain a variety of redox enzymes acting on oligosaccharides and monosugars that are released from cellulose or hemicellulose, as indicated in the “Cellulose depolymerization” panel. Also note that some LPMOs and EGs can act on the hemicellulose fraction, as indicated in the “Hemicellulose depolymerization” panel. A more comprehensive variant of this figure can be found in [39], and a more complete list of enzyme types is provided in Tables 1 and 2: *BG* β-glucosidase, *CBHI* cellobiohydrolase I, *CBHII* cellobiohydrolase II, *CDH* cellobiose dehydrogenase, *EG* endoglucanase, *LPMO* lytic polysaccharide monooxygenase, *RE* redox enzyme (oxidases and peroxidases)

In 1950, Reese et al. postulated that cellulose is degraded in a two-step process, the first step being the conversion of native, crystalline cellulose to shorter, accessible cellulose chains by a component called C_1_ and the second step being the conversion of the now more accessible cellulose to oligomers and monomers by a component called C_x_ [291]. Over the years, the quest towards the isolation of the C_1_ and C_x_ components from fungal secretomes (e.g., [130, 385]) led to the identification of the core set of fungal cellulose-active glycoside hydrolases (GHs), including cellobiohydrolases (CBHs; cleaving off cellobiose from the cellulose chain ends), endoglucanases (EGs; cleaving cellulose chains in non-crystalline regions) and β-glucosidases (BGs; depolymerizing soluble cello-oligosaccharides liberated by CBHs and EGs) [386] (Fig. 2; Table 1). These GHs have been classified, based on sequence similarities, in the Carbohydrate Active enZymes (CAZy) database [219]. As an example, the model organism *T. reesei*, named after one of the pioneers of cellulase research, Elwyn T. Reese, secretes two CBHs, *Tr*Cel7A (formerly CBH I; a reducing end-specific CBH belonging to family GH7) and *Tr*Cel6A (formerly CBH II; a non-reducing end-specific CBH belonging to family GH6), four EGs, named *Tr*Cel7B (formerly EG I), *Tr*Cel5A (formerly EG II or, in the very early days, also EG III), *Tr*Cel12A (formerly EG III), *Tr*Cel45A (formerly EG V) and four BGs, *Tr*Cel3A (formerly Bgl1), *Tr*Cel3B, *Tr*Cel3F and *Tr*Cel3G [1, 231]. Two additional enzymes in the *T. reesei* secretome were initially annotated as EGs, namely *Tr*Cel61A (originally EG IV) [172] and *Tr*Cel61B (originally EG VII), but it is now clear that these enzymes are not EGs but lytic polysaccharide monooxygenases (LPMOs), as discussed below.

**Table 1:** Plant cell wall polysaccharide-active enzymes of fungal origin that may be present in cellulase cocktails

Enzyme name	CAZy	EC	Mode of action	Example^i^
Cellulases				
Cellobiohydrolase (CBH)	GH7	3.2.1.176	Cleaving off cellobiose from the reducing end of cellulose chains	*Tr*Cel7A from *T. reesei* [231]
	GH6	3.2.1.91	Cleaving off cellobiose from the non-reducing end of cellulose chains	*Tr*Cel6A from *T. reesei* [231]
Endo-β-1,4-glucanase (EG)	GH5	3.2.1.4	Cleaving β-(1 → 4)-linkages in cellulose chains in non-crystalline regions (activity on hemicelluloses has been observed for some)	*Tr*Cel5A from *T. reesei* [231]
	GH7			*Tr*Cel7B from *T. reesei* [231]
	GH12			*Tr*Cel12A from *T. reesei* [231]
	GH45			*Tr*Cel45A from *T. reesei* [231]
β-glucosidase (BG)	GH3	3.2.1.21	Cleaving off d-glucose from the non-reducing end of oligosaccharides	*Tr*Cel3A (Bgl1) from *T. reesei* [231]
Hemicellulases				
Xyloglucanase	GH12 (EG)^a^	3.2.1.151	Cleaving β-(1 → 4)-linkages in xyloglucan chains	*Tr*Cel12A from *T. reesei* [393]
	GH74			*Tr*Cel74A from *T. reesei* [231]
Endo-β-1,4-xylanase	GH10	3.2.1.8	Cleaving β-(1 → 4)-linkages in xylan chains	*Tr*Xyn10A from *T. reesei* [231]
	GH11			*Tr*Xyn11A from *T. reesei* [231]
	GH7 (EG)^a^			*Tr*Cel7B from *T. reesei* [15]
Endo-β-1,4-mannanase	GH5	3.2.1.78	Cleaving β-(1 → 4)-linkages in glucomannan main chain	*Tr*Man5A from *T. reesei* [339]
	GH26			*Pa*Man26A *P. anserina* [69]
	GH134			*An*Man134A from *A. nidulans* [319]
	GH5 (EG)^a^			*Tr*Cel5A from *T. reesei* [173]
	GH7 (EG)^a^			*Tr*Cel7B from *T. reesei* [173]
	GH45 (EG)^a^			*Tr*Cel45A from *T. reesei* [173]
β-xylosidase	GH3	3.2.1.37	Cleaving off unsubstituted d-xylose from the non-reducing end of xylo-oligosaccharides	*Tr*Xyl3A (Bxl1) from *T. reesei* [231]
β-mannosidase	GH2	3.2.1.25	Cleaving off unsubstituted d-mannose from the non-reducing end of glucomanno-oligosaccharides	*An*Mnd2A from *A. niger* [3]
Hemicellulose debranching enzymes				
α-arabinofuranosidase	GH43	3.2.1.55	Cleaving off l-arabinosyl substitutions from xylans and xylo-oligosaccharides	HiAraF (GH43) from *H. insolens* [332]
	GH51			*An*AbfA (GH51) from *A. niger* [276]
	GH54			*Tr*Abf1 (GH54) from *T. reesei* [229]
	GH62			*Tr*Abf2 (GH62) from *T. reesei* [20]
α-galactosidase	GH27	3.2.1.22	Cleaving off α-(1 → 6)-linked d-galactosyl substitutions from glucomannan and glucomanno-oligosaccharides	*Tr*Agl1 (GH27) from *T. reesei* [228]
	GH36			*Tr*Agl2 (GH36) from *T. reesei* [228]
α-glucuronidase	GH67	3.2.1.139, 3.2.1.131	Cleaving off α-(1 → 2)-linked d-glucuronic acid (3.2.1.139) or 4-*O*-methyl-d-glucuronic acid (3.2.1.131) sidechains of xylans and xylo-oligosaccharides	*At*AguA (GH67) from *A. tubingensis* [85]
	GH115			*Sc*Agu1 (GH115) from *S. commune* [56]
Deacetylases (incl. acetyl xylan esterase and acetyl mannan esterase)	CE1-6 and CE16^b^	3.1.1.6, 3.1.1.72, 3.1.1.–^e^	Hydrolysis of acetyl groups from various positions in xylans and xylo-oligosaccharides (3.1.1.6 and 72) and/or in glucomannans and glucomanno-oligosaccharides (3.1.1.-)	*Tr*Axe1 (CE5) [316] and *Tr*Axe2 (CE16) [214] from *T. reesei*; *Aw*AXE (CE1) from *A. awamori* [187]; *Np*BnaII (CE2) and *Nc*BnaIII (CE3), and *Nc*BnaI (CE6) from *N. patriciarum* [79] *Vv*AXEII (CE4) from *V. volvacea* [218]; *Ao*AGME from *A. oryzae* [341]^j^
Feruloyl esterase	CE1	3.1.1.73, 3.1.1.–^f^	Cleaving off hydroxycinnamoyl groups esterifying arabinosyl substitutions of xylan backbone or lignin	*An*FaeA from *A. niger* [103] *Nc*FaeD from *N. crassa* [354]
Glucuronoyl esterase (GE)	CE15	3.1.1.–^g^	Cleavage of ester bonds between lignin alcohols and (4-*O*-methyl-d-glucuronic acid substitutions of xylan backbone	*Cu*GE from *C. unicolor* [246]
Lytic polysaccharide monooxygenase (LPMO)	AA9	1.14.99.54	Cleavage of cellulose chains with oxidation at the C1 carbon	*Tt*AA9E from *T. terrestris* [134]
		1.14.99.56	Cleavage of cellulose chains with oxidation at the C4 carbon	*Nc*AA9C from *N. crassa* [7]
		1.14.99.54, 1.14.99.56	Cleavage of cellulose chains with oxidation at the C1 or C4 carbon	*Ta*AA9A from *T. aurantiacus* [284]
		1.14.99.–^h^	Oxidative cleavage of β-(1 → 4)-linkages in xyloglucan chains (C1- and/or C4-oxidation)	*Nc*AA9C from *N. crassa* [7] *Ta*AA9A from *T. aurantiacus* [272]
		1.14.99.–^e^	Oxidative cleavage of xylan	*Mt*AA9A (MYCTH_85556) from *M. thermophila* [116]
	AA10^c^	1.14.99.54	Cleavage of cellulose chains with oxidation at the C1 carbon	*Sc*AA10C from *S. coelicolor* [112]^c^
		1.14.99.53	Oxidative cleavage of chitin (C1-oxidation)	*Sm*AA10A from *S. marcescens* [351]^c^
		1.14.99.54, 1.14.99.56, 1.14.99.53	Cleavage of cellulose chains with oxidation at the C1 or C4 carbon and oxidative cleavage of chitin (C1-oxidation)	*Sm*AA10B from *S. coelicolor* [109]^c^
	AA11	1.14.99.53	Oxidative cleavage of chitin (C1-oxidation)	*Ao*AA11 from *A. oryzae* [139]
	AA13	1.14.99.55	Oxidative cleavage of starch	*Nc*AA13 from *N. crassa* [371]
	AA14	1.14.99.–^e^	Oxidative cleavage of xylan	*Pc*AA14B from *P. coccinea* [68]
	AA15^d^	1.14.99.54	Cleavage of cellulose chains with oxidation at the carbon C1	*Td*AA15A from *T. domestica* [304]^d^
	AA16	1.14.99.54	Cleavage of cellulose chains with oxidation of carbon C1	*Aa*AA16 from *A. aculeatus* [105]

The main CAZy families, the EC number and the mode of action regarding plant cell wall degradation are listed for each activity. Oxidoreductases other than LPMOs are listed in Table 2

^a^This enzyme is primarily known as endoglucanase but has a notable and potentially important side activity on hemicellulose

^b^Deacetylases are discussed together because there is variation in reported substrate preference and specificity among deacetylases belonging to the same CE families, and because the substrate preference (e.g., xylan, glucomannan, pectin or chitin) and/or specificity (deacetylation of e.g., xylosyl, glucosyl or mannosyl residues at position 2, 3 or 6) remains to be identified for most deacetylases. Of note, including deacetylases with complementary activities in cellulase cocktails is of high importance

^c^AA10 LPMOs are rarely found in fungi and are included for the sake of completion; none of the putative fungal AA10 LPMOs have been characterized, and the examples all refer to bacterial enzymes

^d^AA15 LPMOs have not been identified in fungi and are included for the sake of completion; the example refers to an arthropod enzyme

^e^EC number not created yet; no provisional EC number

^f^EC number not created yet; provisional EC number: 3.1.1.B10

^g^EC number not created yet; provisional EC number: 3.1.1.B11

^h^EC number not created yet; provisional EC number: 1.14.99.B11

^i^Strain abbreviations: *A. aculeatus*, *Aspergillus aculeatus*; *A. awamori*, *Aspergillus awamori*; *A. nidulans*, *Aspergillus nidulans*; *A. niger*, *Aspergillus niger*; *A. oryzae*, *Aspergillus oryzae*; *A. tubingensis*, *Aspergillus tubingensis*; *C. unicolor*, *Cerrena unicolor*; *H. insolens*, *Humicola insolens*; *M. thermophila*, *Myceliophthora thermophila*; *N. patriciarum*, *Neocallimastix patriciarum*; *N. crassa*, *Neurospora crassa*; *P. anserina*, *Podospora anserina*; *P. coccinea*, *Pycnoporus coccinea*; *S. coelicolor*, *Streptomyces coelicolor*; *S. marcescens*, *Serratia marcescens*; *S. commune*, *Schizophyllum commune*; *T. aurantiacus*, *Thermoascus aurantiacus*; *T. domestica*, *Thermobia domestica*; *T. reesei*, *Trichoderma reesei*; *T. terrestris*, *Thielavia terrestris*; *V. volvacea*, *Volvariella volvacea*

^j^The CAZy family for this enzyme has yet to be identified

**Fig. 3:**
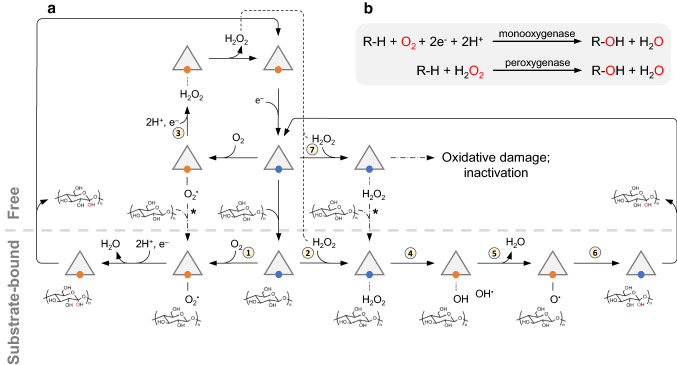
Possible reaction schemes for LPMO-catalyzed cleavage of glycosidic bonds. The triangles represent the LPMO, and the small spheres the active-site copper. Orange spheres depict Cu(II) and blue spheres depict Cu(I). The bottom left of panel **a** shows the O_2_-dependent monooxygenase reaction (1) and the bottom right of panel **a** shows the H_2_O_2_-dependent peroxygenase reaction (2). The upper part of panel **a** shows reactions that may occur in the absence of a polysaccharide substrate. The order of binding events is not fully resolved and the figure shows two scenarios, where the less likely one is labeled by an asterisk. Current data support formation of a ternary complex and do not support a ping-pong mechanism [163, 200]. It is interesting to note that reduction of the LPMO promotes substrate binding [188, 201] and could thus promote ternary complex formation. A scenario where the LPMO remains closely associated with the substrate in between consecutive catalytic cycles is conceivable. Panel **b** shows the simplified reaction schemes for the proposed LPMO reactions. Note that several reaction mechanisms have been proposed for both the monoxygenase reaction [28, 235, 374] and the peroxygenase reaction [37] and that the figure shows one of several possible scenarios for each reaction. The figure also shows the uncoupling reaction with O_2_ that leads to formation of H_2_O_2_ (3; top left). In the H_2_O_2_-dependent reaction mechanism, step 4 indicates homolytic cleavage of the O–O bond of H_2_O_2_, for which experimental and computational evidence is available [38, 163, 375]. One possible outcome is the subsequent formation of an oxyl intermediate (step 5), which has often been proposed as the hydrogen-abstracting intermediate in studies on LPMO catalysis. In this case, hydrogen abstraction would be followed by binding of the resulting hydroxyl to the substrate radical, in an oxygen-rebound mechanism (step 6). Hydroxylation leads to destabilization of the glycosidic bond and will be followed by spontaneous bond cleavage ([274]; not shown). While homolytic cleavage of H_2_O_2_ is supported by recent experimental evidence [163], alternative scenarios are thinkable [37, 163, 375]. Step 7 shows the reaction of a reduced LPMO with H_2_O_2_ in the absence of substrate (top right), which can damage the enzyme and lead to inactivation. It is worth noting that there is at least one additional example of an enzyme, in this case a non-heme mono-iron epoxidase, that was originally thought to be an oxidase (i.e., using O_2_) and that later turned out to use H_2_O_2_ [376]

Although there have been some early indications that oxidative processes contribute to cellulose conversion [99], cellulose decomposition was thought, for a long time, to occur primarily through the action of hydrolytic enzymes. The breakthrough came in 2010 with the discovery of oxidative polysaccharide degradation by enzymes that were previously classified as CBM33s (chitin-binding proteins in bacteria) and GH61s (EGs in fungi) [351]. Today these enzymes are called lytic polysaccharide monooxygenases (LPMOs) and have been reclassified as Auxiliary Activity (AA) families 10 and 9, respectively, in the CAZy database [212]. Over the past decade, several LPMO families have been described, and, as of today, families AA9-11, AA13-14 and AA16 comprise fungal LPMOs. AA15 type LPMOs have not been identified in fungi. Fungal LPMOs of the AA10 type are very rare and, while bacterial AA10s have been intensely studied, none of the putative fungal AA10s have been characterized. LPMOs contain a single copper co-factor, the reduction of which is crucial for the LPMO reaction [284, 351]. These enzymes catalyze the oxidative cleavage of β-1,4-glycosidic bonds of recalcitrant polysaccharides, either in a monooxygenase reaction using molecular O_2_ and a reductant [351] or in a peroxygenase reaction using H_2_O_2_ [37, 38] (Fig. 3).

Importantly, the monooxygenase paradigm entails that reducing equivalents are being consumed by the LPMO in each catalytic cycle, whereas the peroxygenase reaction only requires priming amounts of reductant to reduce the LPMO to its catalytically active Cu(I) state (Fig. 3). It has been shown that the reducing power needed by LPMOs can be delivered in many ways, including a wide variety of small molecule reductants, such as ascorbic acid [351], phenolic compounds, including compounds derived from lignin and plant biomass in general [114, 190, 381], as well as certain redox enzymes [121, 190, 206, 274] (as reviewed by [39, 117]). Both the catalytic mechanism of LPMOs and the relative importance of the O_2_-driven and H_2_O_2_-driven reactions are the subject of debate and current research, as recently reviewed in [39, 60].

Since the postulation of the C_1_–C_x_ theory for cellulose depolymerization by Reese et al. [291], the nature of the C_1_ factor has been interpreted in a number of ways. First, cellobiohydrolases were thought to act as C_1_ factor [129]. It has been suggested that CBHs break non-covalent linkages between adjacent cellulose chains in crystalline cellulose since they thread a single cellulose chain into their active site cleft (or even tunnel) and, thus, are potentially capable of extracting a longer piece of cellulose chain out of its crystalline context [122, 182]. While lifting a single cellulose chain (likely 6 or more glucose units) away from the crystalline lattice, i.e., decrystallization of cellulose, carries an energy penalty, strong binding interactions between the enzyme and the cellulose, which relate to the processive nature of CBHs, could make such decrystallization energetically possible (see also below). Later, Arantes and Saddler proposed that carbohydrate-binding modules (CBMs), such as the one attached to the most studied CBH, *Tr*Cel7A, and expansin-like proteins, such as the Swo1 swollenin protein that induces swelling of cellulose [305], may fulfil the role of the C_1_ factor [10]. The discovery of LPMOs has led to the speculation that these enzymes may in fact be the long-sought-after C_1_ factor [142, 245, 351]. This hypothesis is supported by multiple studies showing that LPMOs belonging to various AA families induce fibrillation of cellulose fibers [149, 352, 364].

Of the *T. reesei* cellulases, the CBH *Tr*Cel7A has gained the most attention, primarily because it is the most abundant enzyme in the secretome, comprising close to 60% of the cellulolytic proteins [126]. The crystal structure of the catalytic domain of *Tr*Cel7A reveals a tunnel-shaped active site [89], which can accommodate ten glucosyl units [64, 88]. The long substrate-binding tunnel of *Tr*Cel7A enables strong interactions with a single cellulose chain and contributes to the processive mode of action of this enzyme [26, 181, 182], as visualized by Igarashi et al. using high-speed atomic force microscopy [151]. Processivity is a key attribute of CBHs that makes them especially powerful in depolymerizing the highly compact structure of crystalline cellulose [26, 338, 362]. On the other hand, processivity leads to stalling of CBHs when their path is blocked by other enzymes or substrate-derived obstacles [73, 113, 152, 155, 199]. Furthermore, it has been claimed that the strong binding energies associated with processivity, in particular reflected in low off-rates [74, 198], make processive GHs intrinsically slow, as has been nicely demonstrated for processive chitinases [141, 394, 395].

Contrary to the CBHs, with their deep substrate-binding clefts, or even tunnels, cellulose-active LPMOs have a flat substrate-binding and catalytic surface, which is optimized for attacking surfaces such as those found in cellulose crystals [171, 350, 351]. Unlike CBHs and other GHs, LPMOs cannot use binding energy to distort the substrate towards the transition state for hydrolytic glycoside bond cleavage. Thus, LPMOs employ powerful oxidative chemistry, allowing them to cleave the β-1,4-glycosidic bonds of cellulose without the need to remove a cellulose chain from the crystalline lattice. Some LPMOs are known to act on non-crystalline substrates [7, 102, 154], and the most commonly used substrate for assaying the activity of cellulose-active LPMOs is phosphoric-acid swollen (so, non-crystalline) cellulose. Still, the ability of LPMOs to attack crystalline and other recalcitrant and insoluble polysaccharide structures [68] is well documented [96, 351, 364] and likely comprises the most important role of these enzymes in biomass conversion.

### Hemicellulolytic enzymes

Depending on the type of biomass and pretreatment technology, pretreated biomass contains, in addition to cellulose, varying amounts of linear and branched polysaccharides, including the hemicelluloses xylan, glucomannan and xyloglucan, as well as pectin, all of which adhere to cellulose fibers, forming a complex three-dimensional matrix [323]. These polysaccharides can form multiple substructures, and while many hemicelluloses are relatively easy to degrade, a fraction of these polysaccharides will form recalcitrant co-polymeric substructures that may hamper cellulose degradation [47, 261, 392]. Due to the high complexity of these plant polysaccharides, a variety of enzyme activities are needed for their complete breakdown (Table 1). The most studied hemicellulose-active enzymes are xylan- and glucomannan-specific enzymes. These hemicellulases include GHs that cleave the polysaccharide main chain, i.e. endo-β-1,4-xylanases (shortly xylanases) and endo-β-1,4-mannanases (shortly mannanases), as well as debranching enzymes that remove substitutions from the polysaccharide backbone (e.g., deacetylases, arabinosidases and galactosidases). These enzymes and their potential uses have been reviewed by Malgas et al. [224, 227]. Interestingly, recent studies indicate that LPMOs belonging to class AA14 may be tailored to specifically act on recalcitrant xylan coating cellulose fibers [68] (Fig. 2).

In addition to hemicellulases, some EGs and AA9 LPMOs may also contribute to hemicellulose conversion because they are capable of cleaving the polysaccharide backbones of some, or even a wide range, of hemicellulosic polysaccharides, including xyloglucan, xylan and/or glucomannan [7, 102, 116, 150, 183, 320, 366] (Fig. 2, Table 1). While promiscuous endoglucanases [366] and some of the hemicellulolytic LPMOs cleaving mixed-linkage glucans, xyloglucan and glucomannan [7, 102, 183, 251, 272, 320], are active on isolated hemicelluloses, xylan-active AA9 (and also AA14) LPMOs [68, 114, 116, 150] require xylan being complexed with cellulose. A likely reason for this is that insoluble forms of hemicelluloses associated with cellulose adopt different conformations than their soluble forms [47]. Consequently, screening for enzyme activity on natural substrates or pretreated biomass instead of model substrates, such as microcrystalline or amorphous cellulose and isolated hemicelluloses, may be a prerequisite for accurately describing substrate specificities, or for detecting enzyme activity in the first place [68].

An evolutionary advantage for substrate promiscuity for EGs and LPMOs could be the ability to cleave recalcitrant fractions of xyloglucan, xylan and glucomannan that adhere to cellulose fibers. As an example, *Tr*Cel7B is active on xylan [15], glucomannan [239] and xyloglucan [366]. In terms of promiscuity among EGs and LPMOs, the fact that GH7 EGs (such as *Tr*Cel7B), and potentially also some AA9 LPMOs, can act on both xylan and glucomannan likely contributes to their importance in enzyme cocktails for biomass breakdown [61, 168, 300, 355]. It is noteworthy that the activity of *Tr*Cel7B from *T. reesei* on xylan is comparable to, if not higher than, its activity on cellulose [15]. Xylans are abundant in all types of lignocellulosic plant biomass (i.e., grasses, hardwood and softwood), emphasizing the importance of xylan-active EGs and CAZymes in general in enzyme cocktails, irrespective of the origin of the feedstock. Most importantly, inclusion of CAZymes with broad substrate specificities will help in designing universal enzyme cocktails for the breakdown of a broad range of biomass.

Complementarily to the action of enzymes converting hemicellulose polymers to shorter fragments, debranching enzymes are needed to enable the complete saccharification of hemicellulosic oligomers by β-xylosidases and β-mannosidases [224, 227]. Some debranching enzyme activities may be of particular importance as they cleave covalent linkages to lignin [157]. Substitutions of xylans include hydroxycinnamoyl and glucuronoyl groups, which have been shown to take part in the formation of covalent linkages between lignin and xylan. Enzymes potentially acting on lignin–hemicellulose bonds include feruloyl esterases, cleaving off hydroxycinnamoyl (including feruloyl, *p*-coumaroyl, and cinnamoyl) groups from arabinosyl substitutions of the xylan backbone [71], and glucuronoyl esterases, cleaving off lignin alcohols having ester bonds with (methyl)-glucuronic acid substitutions of the xylan backbone [101, 243, 246]. These enzymes have received considerable attention as enzymatic cleavage of lignin–polysaccharide bonds potentially has a dual positive effect in biomass conversion: (1) improvement of enzymatic accessibility of plant cell wall polysaccharides and (2) removal of hemicellulose moieties from the residual lignin. The relevance of these enzymes for complete biomass saccharification is emphasized in a recent study by Mosbech et al., showing that a glucuronoyl esterase from *Cerrena unicolor*, in combination with a GH10 xylanase, is able to completely remove xylan moieties from birchwood lignin [246].

Debranching enzymes and deacetylases are especially important in biomass decomposition because hemicelluloses coating cellulose microfibrils, in particular xylan and glucomannan, are known to be acetylated and substituted with glucuronic acid or galactose [46, 125, 392]. Removal of these substitutions changes cellulose–hemicellulose interactions and may decrease the recalcitrance of the feedstock [265]. On the other hand, removal of substitutions from xylan and glucomannan polymers that are not directly associated with cellulose microfibrils may decrease their solubility in water and lead to the adsorption of linear, unsubstituted hemicellulose fragments onto cellulose fibers [165, 195, 379]. While these hemicelluloses can be removed by xylanases and mannanases, they will limit cellulose accessibility [379, 380]. In addition to acting on hemicelluloses, acetyl esterases may also act on lignin and change its properties [265], but the implications of this effect, and of the effects of deacetylating enzymes in general remain to be studied.

### Other oxidoreductases in biomass conversion

In addition to GHs and LPMOs, fungal secretomes are rich in oxidoreductases, including cellobiose dehydrogenases (CDHs; belonging to family AA3_1 in CAZy), lignin-active laccases (family AA1) and peroxidases (family AA2), copper-radical oxidoreductases (family AA5) and multi-copper oxidoreductases (family AA3). A detailed overview of these enzymes and potential interactions between them is provided in a recent review by Bissaro et al. [39]. Some of these oxidoreductases have been shown to directly (CDH) or indirectly (laccase and polyphenol oxidase) interact with LPMOs (Fig. 2; Table 2). CDHs can reduce the active-site copper of LPMOs directly via their AA8 cytochrome domain [335], thus fueling the LPMO reaction, and may also contribute by generation of the LPMO co-substrate H_2_O_2_ [189]. Two polyphenol oxidases have been shown to promote LPMO reactions because they hydroxylate methylated or non-methylated monophenols (including lignin monomers), which thus become better reductants for LPMOs [115]. Alternatively, laccase treatment of lignin, which as such is known to be able to drive LPMO reactions (see above), has led to increased LPMO activity [42, 269]. Perna et al. showed that the observed effect is due to increased H_2_O_2_-production by reactions involving laccase-modified lignin [269]. For the successful exploitation of these effects in biomass conversion, however, further research is needed, addressing, for example, the interaction of lignin-active oxidoreductases with lignin, as well as the actual flow of electrons, the generation and consumption of H_2_O_2_ and effects on both the LPMOs and other enzyme components.

**Table 2:** Fungal oxidoreductases that may be present in commercial cellulase mixtures and that may affect LPMO activity

Enzyme name	CAZy family/EC number	Proposed mode of interaction	Examples^h^
Cellobiose dehydrogenase (CDH)	AA3_1 1.1.99.18	Reduction^a^ and in situ generation of H_2_O_2_^b,c^	*Hi*CDH from *H. insolens* + *Ta*AA9A from *T. aurantiacus* [206] *Mt*CDH-1 from *M. thermophila* + *Tt*AA9E from *T. terrestris*, *Mt*AA9E (MYCTH_85556) from *M. thermophila*, and *Tr*AA9A from *T. reesei* [45] *Mt*CDH-2 from *M. thermophila* + *Nc*AA9M [274], 9D, and 9E [27, 274] and *Nc*AA13 [370] from *N. crassa* and *Mt*AA9E (MYCTH_79765) [131] and variants of *Mt*AA9D (MYCTH_92668) from *M. thermophila* [324] *Mt*CDH from *M. thermophilum* + *Nc*AA9F [335], 9C [43, 154, 273], 9A, and 9D [273] from *N. crassa* and *Ta*AA9A from *T. aurantiacus* [272] *Mt*CDH from *M. thermophilum* + *Sc*AA10C from *S. coelicolor* and *Sm*AA10A [37, 220] and variants thereof [221] from *S. marcescens* Variants of *Mt*CDH from *M. thermophilum* + *Nc*AA9C from *N. crassa* and *Sm*AA10A from *S. marcescens* [189]^c^ *Nc*CDH IIA + *Nc*AA9C [67, 104, 180, 190, 330], 9F [180, 190, 335], 9E, and 9J [180, 190] from *N. crassa* and *Ps*AA9A and 9B from *Pestalotiopsis* sp. [263] *Nc*CDH IIB + *Nc*AA9C [180, 190, 330], 9E, 9F, and 9J [180, 190] from *N. crassa* and *Ps*AA9A and 9B from *Pestalotiopsis* sp [263] *Pa*CDHB + *Pa*AA9A, 9D, 9E, 9F, 9G, and 9H from *P. anserina* [31] *Pc*CDH from *P. cinnabarinus* + *Pa*AA9A and 9B from *P. anserina* [34] *Tt*CDH + *Tt*AA9E from *T. terrestris* [206]
		Reduction of redox mediators that can affect LPMO reactions^d^	The AA3_1 domain of *Mt*CDH from *M. thermophilum* + *Nc*AA9C from *N. crassa* [190]
Pyranose dehydrogenase (PDH), PQQ-dependent	AA12 1.–.–.–	Reduction^a^ and, possibly^e^, in situ generation of H_2_O_2_^f^	*Cc*PDH from *C. cinerea* + *Nc*AA9C and 9F [357] and *Nc*AA9A and 9D [273] from *N. crassa*
Pyranose dehydrogenase (PDH), FAD-dependent	AA3_2 1.1.99.29	Reduction of redox mediators that can affect LPMO reactions^d^	*Am*PDH from *A. meleagris* + *Nc*AA9C from *N. crassa* [190]^i^
Glucose dehydrogenase GDH	AA3_2 1.1.5.9	Reduction of redox mediators that can affect LPMO reactions^d^ and, possibly^e^, in situ generation of H_2_O_2_	GDH from *G. cingulata* + *Nc*AA9C from *N. crassa* [190]^i^ GDH from *P. cinnabarinus* + *Pa*AA9E from *P. anserina* [121]
Glucose 1-oxidase (GOx)	AA3_2 1.1.3.4	Reduction of redox mediators that can affect LPMO reactions^d^	*An*GOx from *A. niger* + *Nc*AA9C from *N. crassa* [190]^i^
		In situ generation of H_2_O_2_^g^	*An*GOx from *A. niger* + *Sc*AA10C from *S. coelicolor* [37] *An*GOx from *A. niger* + *Nc*AA9C from *N. crassa* [104]
Aryl-alcohol quinone oxidoreductase (AAQO)	AA3_2	Reduction^a^ and, possibly^e^, in situ generation of H_2_O_2_	AAQO1 and AAQO2 from *P. cinnabarinus* + *Pa*AA9E from *P. anserina* [121]
Aldose oxidase (AOx)	AA7 1.1.3.-	In situ generation of H_2_O_2_	*Mn*AOx from *M. nivale* + *Ta*AA9A from *T. aurantiacus* and Cellic CTec3 [266]^j^
Laccase	AA1 1.10.3.2	Generation of H_2_O_2_ via lignin oxidation	Laccase from *T. versicolor*, *M. thermophila*, *G. lucidum*, and *Amycolatopsis* sp. + *Sm*AA10A from *S. marcescens* and *Nc*AA9C from *N. crassa* [269]
Polyphenol oxidase	(not in CAZy) 1.14.18.1	Activation of lignin for more efficient reduction^a^ and/or in situ generation of H_2_O_2_^e^	*Ab*PPO from *A. bisporus* and *Mt*PPO7 from *M. thermophila* driving *Mt*AA9B (MYCTH_80312) from *M. thermophila* [115]
Versatile peroxidase	AA2 1.11.1.14	LPMO-generated H_2_O_2_ drives peroxidase activity	*Ps*VP from *Physisporinus* sp. + *Po*LPMO9A from *P. ostreatus* [213]
Catalase	(not in CAZy) 1.11.1.6	Preventing oxidative damage by keeping H_2_O_2_ concentrations low	Catalase from *T. aurantiacus* + *Ta*AA9A from *T. aurantiacus* and Cellic CTec3 [266, 312] Catalase from *C. glutamicum* + *Nc*AA9C from *N. crassa* [104]

The tested enzyme pairs and the (putative) modes of interaction between them are listed for each type of oxidoreductase

^a^The role and nature of the reduction step differs between catalytic scenarios, as outlined in the main text and Fig. 3 [37]. Reduction may be seen as a “priming event”, i.e., activation of the LPMO for subsequent multiple H_2_O_2_-driven turnovers. Alternatively, in the O_2_-driven scenario, two electrons need to be delivered per catalytic cycle

^b^Electron transfer from CDH to the active site copper of the LPMO is mediated by the AA8 cytochrome domain and has been observed in several studies, e.g., [190, 330, 335]. Alternatively, electrons may be transferred directly from the DH domain to O_2_, leading to the generation of H_2_O_2_ [189]

^c^Reference [189] provides evidence showing that the ability of engineered CDH variants to drive LPMO reactions correlates with the ability of these variants to generate H_2_O_2_

^d^The role of redox mediators has been addressed in various studies and has so far only been linked to reduction of the LPMO. Redox mediators may also affect H_2_O_2_ levels in the reaction

^e^The production of H_2_O_2_ and its potential impact on the LPMO were not assessed, but it is conceivable that H_2_O_2_ production occurred under the conditions used

^f^The domain structure of *Cc*PDH is analogous to that of CDHs, suggesting that the two enzymes use similar mechanisms in driving LPMO reactions [357]

^g^GOx can generate H_2_O_2_, the co-substrate of LPMOs, but is unable to reduce LPMOs [37]

^h^Strain abbreviations: *A. bisporus*, *Agaricus bisporus*; *A. meleagris*, *Agaricus meleagris*; *A. niger*, *Aspergillus niger*; *C. cinerea*, *Coprinopsis cinerea*; *C. glutamicum*, *Corynebacterium glutamicum*; *G. cingulata*, *Glomerella cingulata*; *G. lucidum*, *Ganoderma lucidum*; *H. insolens*, *Humicola insolens*; *M. nivale*, *Microdochium nivale*; *M. thermophila*, *Myceliophthora thermophila*; *M. thermophilum*, *Myriococcum thermophilum*; *N. crassa*, *Neurospora crassa*; *P. anserina*, *Podospora anserina*; *P. cinnabarinus*, *Pycnoporus cinnabarinus*; *P. ostreatus*, *Pleurotus ostreatus*; *S. coelicolor*, *Streptomyces coelicolor*; *S. marcescens*, *Serratia marcescens*; *T. aurantiacus*, *Thermoascus aurantiacus*; *T. terrestris*, *Thielavia terrestris*; *T. versicolor*, *Trametes versicolor*

^i^The ability of the enzyme to reduce redox mediators that can affect LPMO reactions was tested; reactions with LPMO, i.e., the enzyme, redox mediator and LPMO, were not shown

^j^While this study showed in situ generation of H_2_O_2_, it did not show a beneficial effect of AOx on LPMO activity

## Co-operativity between enzyme components

**Fig. 4:**
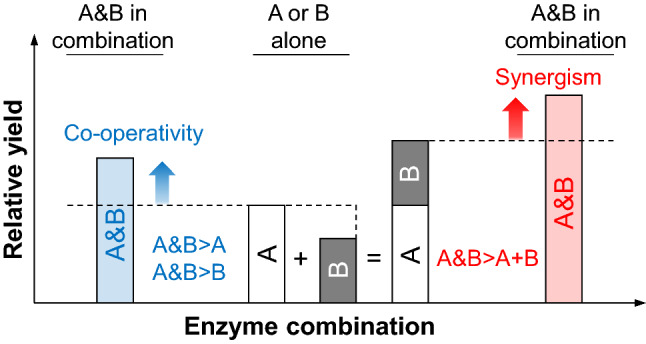
Schematic representation of the difference between co-operativity and synergism between enzymes. Co-operativity between two or more enzymes implies that concomitant action of the enzymes gives saccharification yields that are higher than the yields obtained in reactions with individual enzymes (on the left, in blue). Synergism between enzymes implies that the concomitant action of the enzymes results in a yield that is higher than the sum of the yields obtained in reactions with the individual enzymes (on the right, in red)

In order to gain a deeper understanding of the mechanisms behind enzymatic biomass decomposition, individual enzyme components have been studied alone (enzyme characterization studies) and in combination with other individual enzyme components (minimal enzyme cocktail studies), cellulase cocktails or fungal secretomes (supplementation or spiking studies). Already in the late 1970s, co-operativity (Fig. 4) between different cellulases became clear when Wood and McCrae showed that CBHs enhance swelling of cotton fibers by EGs [387]. Shortly thereafter, CBHs and EGs were described to exert a mutually positive effect on each other’s action during cellulose hydrolysis [140]. In other words, it was demonstrated that these two enzymes act synergistically (Fig. 4). Since then, several types of synergism have been observed between cellulolytic enzymes: between CBHs and EGs [253], CBHs, EGs and cellulose-active AA9 LPMOs [134], and two cellulose-active AA10 LPMOs [109]. The mechanisms of synergies between cellulolytic enzymes have been in the focus of research on biomass degradation, especially for cellulose, using for example detailed kinetic models [155, 253, 373] and atomic force microscopy [96, 120, 152]. A classical interpretation of this synergy is that EGs generate new chain ends for CBHs, but recent studies have indicated that additional mechanisms need to be considered [41, 100, 155, 202, 257, 279]. In particular, it has been proposed that EGs may promote CBH activity by attacking amorphous regions in the cellulose that CBHs are unable to pass during processive action [155, 279].

Over the past decade, the interplay of LPMOs with hydrolases has gained considerable attention [11, 96, 97, 175, 248]. Studies with chitin-active [349] and cellulose-active [134, 238] LPMOs have shown that these enzymes promote the action of classical hydrolytic enzymes, and after the discovery of the catalytic activity of LPMOs [351], it became clear that the presence of reducing power promotes the LPMO effects. Indeed, Harris et al. observed that the boosting effect of an LPMO on cellulase action required the presence of other compounds in the biomass, most likely lignin-derived [134]. In retrospect, it is clear that these observations relate to the reducing power that is present in biomass but not in model cellulosic substrates such as Avicel [134, 143, 247]. In an important study, Eibinger et al. used confocal microscopy to show that a cellulolytic LPMO from *N. crassa* primarily acts on surface-exposed crystalline areas of the cellulose and that LPMO treatment promoted adsorption of a CBH, *Tr*Cel7A, to these crystalline regions, resulting in more efficient hydrolysis of these cellulose crystals [96]. Subsequent studies using real-time atomic force microscopy led to similar conclusions [97]. The work by Eibinger et al. provides evidence that at least some LPMOs cleave cellulose at crystalline areas and thus produce new chain ends, i.e. action sites, for CBHs. This highlights an important difference between LPMOs and EGs in terms of their mode of synergism with CBHs, since these enzymes cleave crystalline and amorphous parts of cellulose, respectively.

Notably, the oxidation at the terminal glucose molecules after LPMO action will have multi-faceted impact on CBHs that will depend partly on the directionality of CBHs and partly on the affinity of individual CBHs for the oxidized chain ends. One of the two new chain ends generated by an LPMO will be oxidized, and CBHs may vary in terms of how well they interact with such oxidized chain ends. Interestingly, molecular simulation studies on the oxidative cleavage of crystalline cellulose by LPMOs performed by Vermaas and colleagues indicated that C4-oxidized chain ends (i.e. oxidized at the non-reducing end) will be more readily hydrolyzed by non-reducing end-specific GH6 CBHs, such as *Tr*Cel6A [361].

Co-operativity between enzymes has also been studied in detail for degradation of various hemicelluloses [83], including xylan [224] and glucomannan [227], the most abundant hemicelluloses in lignocellulosic biomass. On hemicelluloses, synergism occurs primarily between enzymes hydrolyzing the polysaccharide main chain and debranching enzymes. For xylan depolymerization, examples include synergism between the following: a xylanase and an arabinosidase [186, 360], xylanases and a glucuronidase [85], xylanases and acetyl esterases [35], a GH11 xylanase and a CE5 acetylxylan esterase [315, 316], a GH10 or GH11 xylanase and a CE1 feruloyl esterase [84, 103] and a GH10 xylanase and a CE15 glucuronoyl esterase [246]. In addition, synergism between a GH11 xylanase and an AA14 LPMO, both acting on the xylan backbone, has recently been observed [68]. Saccharification of glucomannan has been less studied because the plant cell walls of grasses and herbaceous plants, the more commonly used feedstocks for ethanol production, do not contain glucomannan. Examples of enzyme synergism in glucomannan degradation include the following: a mannanase and galactosidases [63, 228], a mannanase a galactosidase and two acetyl esterases [341], and a GH5 mannanase and a CE2 acetyl esterase [12].

Notably, studies on polysaccharide utilization loci in bacteria from the gut microbiota may provide further insight into the interplay of backbone-cleaving and debranching enzymes for compounds such as xyloglucan [208], pectin [222], xylan [297] and glucomannan [76, 204]. Since these polysaccharide utilization loci likely encode all enzymes needed for saccharification of a certain polysaccharide, they provide hints as to the preferred composition of enzyme cocktails for biomass saccharification containing fungal enzymes.

In natural biomass, cellulose, hemicelluloses (xyloglucan, xylan and/or glucomannan), pectin and lignin co-occur, and hence synergism of enzymes acting on different plant cell wall components can be anticipated to occur. Such “intermolecular synergism” has been described in the late 1990s for cellulases and xylanases acting on birch kraft pulp and for cellulases, xylanases and mannanases acting on spruce kraft pulp by Tenkanen et al. [340] and later for CBH and xylanase acting on pretreated corn stover by Selig et al. [316]. The interplay between cellulases and enzymes acting on hemicellulose has also been extensively studied by the Saddler group [144–146, 327]. Notably, cellulases, xylanases and mannanases work synergistically with each other on spruce chemical pulp not only in the initial phase of the saccharification Várnai [359] but also throughout the course of the reaction. Apparently, depolymerization of cellulose, xylan and glucomannan proceeds simultaneously throughout the process, indicative of a “peeling” type of synergism [355]. In a recent study, Nekiunaite and co-workers showed that cleavage of cellulose by a cellulose-active LPMO from *N. crassa* is inhibited by the presence of xyloglucan and that this inhibition is alleviated by adding a xyloglucan-active EG [251]. These findings point at the possible importance of promiscuous EGs [366] and LPMOs [7, 102, 114, 150] in the complete saccharification of lignocellulosic biomass. It seems clear that for the complete saccharification of any feedstock of interest, it is essential to identify key plant cell wall components that may hinder access to cellulose and other plant cell wall polysaccharides and to identify the corresponding carbohydrate-active enzymes (CAZymes) that cleave these.

### Co-operativity or synergism?

It is important to note that the term synergism should be used with care. Synergism between two enzyme components occurs if the concomitant action of the two enzymes results in a higher yield than when summing up the yields obtained when using the individual components (Fig. 4). Synergism is best observed between pure enzymes using low enzyme dosages and short reaction times, i.e. staying in the initial linear phase of the saccharification reaction [9, 225, 355]. Using longer incubation times may mask positive effects of combining enzymes acting on the same plant cell wall polymer. This can happen when the concomitant action of the enzymes leads to faster saccharification, which can be observed in the initial phase, but does not lead to higher final conversion yields.

While carefully designed laboratory experiments addressing synergistic effects may give insights into the mechanism of interaction between a selection of individual enzyme components, understanding the importance of individual enzyme components in cellulase cocktails remains challenging. To elucidate the effect of individual enzyme components on the total conversion yield, studies on the development of minimal enzyme cocktails (i.e. optimizing blends of individual enzymes [21, 61, 168]) as well as spiking studies (i.e. partial replacement or supplementation of cellulase cocktails with an enzyme preparation [143, 146, 177, 250]) are used routinely. Such studies can lead to the identification of key enzyme components that are necessary for efficient saccharification of a feedstock. Since enzyme production costs (i.e., protein production costs) are an important factor in enzyme-based biorefining, it is important that the total protein loading is fixed in studies aimed at investigating enzyme co-operativity and identification of limiting activities [145]. A few examples of enzyme activities that may be limiting in the industrial conversion of lignocellulosic biomass are discussed below.

### LPMOs and catalases

Using technical substrates (i.e. pretreated biomass) to test the performance of enzyme cocktails is essential for industrial relevance. This is exemplified by the early work of Harris et al., which indicated that LPMOs are active on lignocellulosic substrates (such as pretreated corn stover) but not on pure cellulose substrates [134]. An explanation for these initial findings only became clear after the discovery that LPMOs need electrons, which lignin can provide [114, 381]. Recent studies indicate that lignin has a dual function in LPMO activation: it is able to reduce the active site-copper of LPMOs and to produce H_2_O_2_ in situ from O_2_ [185, 269]. Importantly, lignin-active enzymes can affect the electron-donating and H_2_O_2_-generating abilities of lignin, providing possible links between polysaccharide- and lignin-degrading enzyme systems [42, 115, 269]. Another possible link between these systems is that LPMO-facilitated in situ production of H_2_O_2_ may be utilized by peroxidases to degrade lignin [213].

To employ LPMOs in the degradation of lignin-poor cellulosic substrates, it is necessary to supply the saccharification reaction with external reducing agents like ascorbic acid to activate the LPMOs [250]. For saccharification of cellulose-rich sulfite-pulped spruce, it has been shown that lignin-containing spent sulfite liquor can work as an electron donor [62, 65]. On the other hand, accumulating data confirm that the LPMO reaction can be driven by lignin remaining in the biomass after various pretreatments, including dilute-sulfuric acid pretreatment [134], steam explosion [250] or hydrothermal pretreatment [48, 185], although to varying extents [296]. Thus, while lignin may be inhibitory to cellulases due to unproductive enzyme binding [23, 32, 91, 260, 287, 288, 347] or shielding the polysaccharide [90, 191], it may be crucial for LPMO activity in certain experimental settings.

LPMO activity depends on supply of H_2_O_2_, either direct or indirect, i.e in situ production of H_2_O_2_ from O_2_. The latter needs a much higher supply of reductant (Fig. 3) and may only be feasible when the feedstock is relatively rich in lignin. For substrates with low lignin content, direct supply of H_2_O_2_ works extremely well [248], also at demonstration scale [65]. For lignin-rich substrates, however, the benefits of direct addition of external H_2_O_2_ are less clear [248], presumably due to side-reactions occurring between added H_2_O_2_ and lignin [185]. In situ production of H_2_O_2_ may happen close to the enzyme, perhaps even on the enzyme, which will increase the likeliness that the generated H_2_O_2_ is indeed used by the LPMO rather than being consumed in side reactions between H_2_O_2_ and lignin.

A drawback of processes relying on in situ production of H_2_O_2_ is the lack of direct control over the amount of H_2_O_2_ produced, meaning that intermittently high concentrations of H_2_O_2_ (and other reactive oxygen species derived from H_2_O_2_) could be experienced, which may be damaging to the enzymes. Accumulation of H_2_O_2_ may be prevented by the use of catalases, which convert H_2_O_2_ to water. Indeed, a study by Scott et al. showed that inactivation of LPMO-containing cellulase blends was significantly reduced by addition of catalases [312]. Thus, a likely role of catalases, which are also present in fungal secretomes together with LPMOs [2], is to maintain low H_2_O_2_ levels in systems with in situ H_2_O_2_ generation (Table 2). Since catalases have *K*_m_ values for H_2_O_2_ in the millimolar range, while LPMOs have *K*_m_ values for H_2_O_2_ in the micromolar range [39, 200], LPMOs will still be active and not directly inhibited by the H_2_O_2_ consumption of the catalases. It should also be noted that abiotic factors will consume oxygen and generated reactive oxygen species during typical incubation conditions for enzymatic saccharification of lignocellulosic materials (as illustrated in Fig. 2), and many aspects of the reactions taking place are not yet fully understood [266].

## Today’s cellulase cocktails: what are the limitations and how to overcome these?

Commercial enzyme cocktails have been greatly improved since initial cocktails were launched on the market [160, 238]. Most commercial cocktails are fungal-derived because several fungi are efficient degraders of plant biomass and show high production levels of catalytically efficient cellulases. Family GH7 cellulases are generally considered to be highly efficient and are only found in fungi. Fungi secrete lignocellulose-degrading enzymes into the medium, enabling easy separation from the producing organism Merino and Cherry [238]. However, fungal secretome profiles differ between fungal strains and may vary a lot depending on the carbon source [2, 30, 59, 240, 278]. This must be carefully considered when trying to select natural enzymes for conversion of differently pretreated biomass feedstocks. Despite a lack of publicly available data, it is clear that optimization of enzyme cocktails will have different outcomes for different feedstocks and that a one-size-fits-all strategy may not be optimal [33, 136].

Through the years, individual components of the enzyme cocktails have been the subject of enzyme improvement [268], either through screening for novel enzymes from alternative organisms (e.g., [133, 299, 326]) or by applying enzyme engineering technologies (e.g., [6, 80, 244, 313]). Work done on commercial enzymes is not generally known to the public; typical targets for improvement of individual cellulases include increased hydrolytic efficiency and/or stability at process conditions, reduced end-product inhibition and reduced lignin binding. Enzyme engineering strategies include directed evolution, usually based on combining random and site-directed mutagenesis steps [124, 244, 368], modification of the linker region of bimodular cellulases [14, 313] and domain shuffling, i.e., creation of fusion/chimeric proteins by combining (partial or complete sequences of) catalytic domains and CBMs from different enzymes/organisms [138, 331, 337, 369]. Despite the tremendous work that has been done for cellulase optimization, we are still trying to understand certain fundamentals of how EGs and CBHs work, and work together, the aim being to develop better (mixtures of) EGs and CBHs [176, 203, 257, 303, 362].

The significance of BG activity in alleviating end-product inhibition of CBHs by cellobiose accumulating during lignocellulose conversion was already clear in the late 1970s [325]. Sternberg et al. [325] showed that *Aspergillus* secretomes contain high levels of BG and can be used to compensate for the insufficient levels of BG activity in *Trichoderma* secretomes. In an early and quite unique study, Nieves et al. [254] assessed 13 commercial enzyme preparations from seven companies, including Novozymes’ Celluclast 1.5L derived from *T. reesei*, for cellulolytic (i.e. filter paper) and β-d-glucosidase activities. The results of this study confirmed that the ratio of β-glucosidase-to-cellulase activity was two orders of magnitude higher in the *A. niger* preparations than in the *T. reesei* preparations. Novozymes’ Celluclast 1.5L had the lowest BG titer of the tested *T. reesei* cocktails. A more recent report by Merino and Cherry [238] from Novozymes Inc. showed that engineering the production strain for Celluclast 1.5L to express a BG from *A. oryzae* led to significant improvement in both the conversion yield and rate of cellulose saccharification by the cellulase preparation. Notably, cellulase cocktails that were subsequently launched on the market, including Novozymes’ Cellubrix or Cellic CTec series, have increased BG activity [48, 166] and do not require supplementation with BG for obtaining maximum saccharification efficiency, indicating that the production strains have been developed to express BGs at sufficiently high levels. Novozymes have recently discontinued the sales of their BG product Novozym 188, which has been commonly used to supplement Celluclast 1.5L.

While the oxidative mechanism of LPMOs was not uncovered until 2010 [351], it was already clear in 2007 that these proteins, at the time classified into the GH61 family, had the potential to improve hydrolysis yields by *T. reesei*-produced cellulase cocktails. Merino and Cherry [238] observed that addition of certain *T. terrestris*-produced GH61s at less than 5% of the total protein load in hydrolysis reactions with Celluclast 1.5L enabled reductions in the total enzyme loading by up to two times. Similarly to BGs, GH61s, today called LPMOs, have been incorporated in the Cellic CTec series [48, 62, 135, 160, 250]. Of note, while the contribution of LPMOs to the efficiency of today’s cellulase cocktails is clear and important [49, 65, 146, 167, 248–250], optimizing this impact is not easy and requires careful consideration of reaction conditions [60], as discussed below.

Depending on the substrate pretreatment method, hemicellulases may also play a critical role in lignocellulose depolymerization. When working with substrates pretreated using neutral or alkaline conditions, hemicellulases may be of particular importance as these methods often leave hemicellulose fractions more or less intact Merino and Cherry [238]. It is well established that xylanase supplementation enhances cellulose conversion in biomass prepared by leading pretreatment methods, such as AFEX, ARP and dilute acid treatments, and that this effect is due to the removal of insoluble xylan, which limits cellulose accessibility [196]. Xylanases may also contribute by conversion of soluble xylo-oligosaccharides, which can inhibit cellulases [242, 283] to monomers. A study by Hu et al. on saccharification of steam-pretreated corn stover and poplar showed that, in addition to LPMOs, xylanases contribute to the efficiency of Cellic CTec2 [146]. As another example, the data sheet for Dupont’s Accellerase Trio shows that this cellulase preparation is enriched in xylanases [94]. To cope with the variation of hemicellulose types and contents in a broad range of industrial biomasses, enzyme companies have developed hemicellulolytic preparations (e.g., Novozymes’ Cellic HTec, DuPont’s Accellerase XC, Genencor’s Multifect Xylanase, Dyadic’s FibreZyme, and AB Vista’s Econase XT) that may be used to supplement base cellulolytic preparations (e.g., Novozymes’ Cellic CTec or DuPont’s Accellerase 1500). Notably, lignocellulosic ethanol plants primarily work with grasses, e.g., bagasse, corn stover and giant reed, which contain high amounts of xylans but lack glucomannan. With the exploration of other potential feedstocks, including hardwood and especially softwood biomass, which contain other types of hemicelluloses, further improvement of enzyme cocktails on this front is likely needed (see below).

### Improvement of fungal strains for production of monocomponent enzymes and enzyme cocktails

As recently reviewed by Bischof et al. [36], *Trichoderma reesei* was discovered by researchers at the Natick Army Research Laboratories during World War II. Screening of 14,000 moulds isolated from rotting cellulose-based army equipment in the Solomon Islands for the ability to degrade crystalline cellulose resulted in the identification of the renowned ancestor of all current commercial *T. reesei* strains, designated as QM6a. Random mutagenesis of the *T. reesei* strain QM6a at Rutgers University led to the *T. reesei* strain RUT-C30, which is the prototype hyperproducer of cellulases and is commercially available [36, 271]. One of the key breakthroughs was truncation of the CRE1 transcription factor responsible for repressing the transcription of cellulase genes in the presence of glucose, which led to a substantial increase in cellulase production [236]. Decades of genetic engineering of *T. reesei* has resulted in detailed knowledge of regulators and transcription factors involved in enzyme expression, which again has contributed to the generation of novel cellulase hyperproducing mutants, as reviewed by Bischof et al. [36]. Alternative to genetic engineering of transcriptional regulators, other approaches to enhance expression levels of lignocellulose-active enzymes in *T. reesei* entail understanding the external conditions that affect transcription and expression levels in fungal hosts [314], as well as promoter engineering, epigenetic engineering and metabolic engineering [92].

While *T. reesei* has played a vital role in the history of understanding and exploiting natural lignocellulose-degrading enzyme systems, other filamentous fungal species, including *Aspergillus* sp. [82], *Neurospora crassa* [93] and *Myceliophthora thermophila* [365], have also been studied in detail and may provide useful sources of enzymes or be developed as expression hosts for production of monocomponent enzymes or cellulase cocktails. Expression of recombinant proteins in filamentous fungi is traditionally based on the use of native expression systems, using innate transcriptional regulators and promoters. Transcriptional regulatory systems have been extensively studied in a wide variety of filamentous fungi [106, 241], and it has become clear that these systems are not widely conserved. Hence, knowledge of these systems is often not transferrable from one host organism to another, which is one of the reasons why the development of new filamentous fungal expression hosts is relatively slow [106, 241]. For species such as *T. reesei*, *A. niger* and *A. oryzae*, important regulatory systems are well-explored, as recently reviewed by Mojzita et al. [241]. In addition, relevant transcriptional regulators have been studied to varying extents for *N. crassa* [70, 197], *M. thermophila* [365, 378] and *Thermoascus aurantiacus* [309].

For the production of monocomponent enzymes, the target gene is commonly expressed under a strong promoter [22, 58, 106, 282, 365]. In some cases, rational engineering of the promoter may be used to enhance selective production of a recombinant protein in filamentous fungi; however, this approach is complex and often requires large-scale changes to entire gene networks [106]. Synthetic promoters are currently being considered more promising, since these can contribute to metabolism-independent protein expression [290]. Interestingly, external environmental factors such as light may affect the expression of plant cell wall-degrading proteins in filamentous fungi [308] and such factors thus need to be considered. A recent review on the use of light-regulated promoters addresses the potential of using external environmental factors to induce expression of heterologous proteins in filamentous fungi [118].

Additional strategies for improving fungal production of heterologous proteins include introducing multiple copies of the gene of interest into the expression host [390], fusing target genes to innate genes that are strongly transcribed and developing protease-deficient strains [75]. Most importantly, fungal strain development also includes the production of strains with low (hemi)cellulolytic background tailored for production of single enzymes or completely defined enzyme cocktails. Current industrial strains include Novozymes Inc.’ protease-deficient *A. oryzae* JaL250 strain [390] as well as Roal Oy’s cellulase-deficient *T. reesei* strain [329], DSM’s cellulase-deficient *T. reesei* strain [5] and DSM’s protease- and (hemi)cellulase-deficient *M. thermophila* (previously *Chrysosporium lucknowense*) LC strain [281, 365]. Of note, these strains are the results of major (commercial) research investments and are not publicly available.

Recent work by Steven Singer and co-workers has demonstrated that *T. aurantiacus* has a promising potential to become a thermophilic fungal expression host. *T. aurantiacus* secretes a limited number of endogenous plant cell wall-degrading enzymes, and the natural secretome, despite being relatively simple, has high efficiency in biomass hydrolysis [233, 309]. As a first step, the Singer team has shown that xylose acts as an inducer for production of both cellulases and xylanases in *T. aurantiacus* [310] and has identified related regulatory elements, homologues of which occur in the genomes of other Ascomycetes [309].

While traditional strain development of fungal strains is tedious and time-consuming, the availability of an ever-expanding number of fungal genome sequences through the Joint Genome Institute’s 1000 Fungal Genomes Project [162] and advanced gene-editing technologies [289] together enable the development of alternative fungal enzyme factories. Novel CRISPR/Cas9-based tools will facilitate the development of a variety of novel fungal hosts for heterologous protein production. Indeed, CRISPR/Cas9 has already been adapted successfully to engineer cellulase hyper-producing strains of *Myceliophthora* species [217] and to recombinantly express enzymes in filamentous fungal hosts [290].

### Identification of missing and underperforming enzyme components

Depending on the type of biomass and pretreatment technology, pretreated biomass feedstocks differ in composition and structure and thus hydrolysability by the same cellulase preparation, indicating the need for tailoring enzyme cocktails to the feedstock [143, 196, 318]. In addition to chemical composition and substrate structure, the soluble fraction of pretreated biomass, containing xylo-oligosaccharides and water-soluble lignin degradation products, may restrict the efficiency of some enzymes, due to inhibitory effects, while it may boost the efficiency of others, in particular LPMOs [226, 283, 381, 396]. Detailed studies have confirmed that the type of pretreatment impacts the efficiency of individual enzyme components, such as the CBH *Tr*Cel7A from *Hypocrea jecorina* (anamorph *T. reesei*) [159] and the LPMO *Ta*AA9A from *T. aurantiacus* [143], which, in turn, affects the optimal composition of the enzyme cocktail necessary for breaking down the feedstock [144]. Therefore, the use of industrially relevant pretreated substrates is a prerequisite when evaluating the efficiency of enzyme cocktails and when trying to identify key enzyme activities that may be missing or underrepresented in the enzyme cocktail.

#### What have we learnt from minimal enzyme cocktail studies?

As a first approximation, optimizing the composition of a core set of cellulases, possibly also including one or more hemicellulases, for maximizing saccharification of pretreated feedstock gives good indications as to which enzyme components are important. In general, minimal enzyme cocktail studies have confirmed that there is no “one-fits-all” enzyme cocktail and that the ratio of enzyme components in the optimized mixture depends both on the type of biomass and pretreatment [21, 168, 174]. As an example, mannanases are not required for the saccharification of grasses, such as corn stover, which contain no glucomannan, while mannanase activity is essential for the saccharification of pretreated feedstocks that contain < 2% (even as low as 0.2%, w/w) glucomannan [21, 355]. In another study, Chylenski et al. showed that a four-component enzyme mixture that consists of *Tr*Cel7A and *Tr*Cel6A (CBHs), *Tr*Cel7B (EG) and *An*Cel3A (BG) and that had been optimized for degradation of sulfite-pretreated spruce was equally or more efficient than Cellic CTec2 and CTec3 [61]. Analysis of the hemicellulase activities of the optimized and commercial enzyme mixtures indicated that the efficiency of the minimal enzyme mixture on spruce most likely stems from its higher activity against glucomannan as compared with the commercial preparations. It is well known that *Tr*Cel7B can not only act on cellulose but also on glucomannan [173, 239].

Importantly, three independent studies have found that the proportion of the xylan-active EG *Tr*Cel7B (19–30%, w/w) is significantly more important than that of another EG, *Tr*Cel5A (0–2%, w/w), in enzyme mixtures optimized for saccharification of pretreated barley straw, corn stover and wheat straw [21, 168, 300]. When optimizing a 16-component *T. reesei* enzyme mixture for the saccharification of AFEX-treated corn stover, Banerjee et al. found that *Tr*Cel7A, *Tr*Cel7B, *Tr*Cel61A (= *Tr*AA9A), *Tr*Xyn11A, and *Tr*Xyn10A and the *Tr*Cel3A BG were the most important components [21], emphasizing the importance and complementarity of processive CBHs, promiscuous (i.e., xylan-active) EGs, LPMOs and xylanases for complete biomass degradation. Notably, only a handful of studies included LPMOs in their enzyme mixtures [21, 61, 87, 174]. The results of these studies indicate a correlation between the lignin content of the pretreated feedstock and the importance of LPMO in the enzyme mixture, which may be attributed to the ability of lignin to drive LPMO reaction, as discussed above (e.g., [185, 381]). When assessing the optimal proportion of LPMO in the enzyme mix, process conditions will have to be taken into account, too, since the LPMO reaction requires a source of oxygen.

While most minimal enzyme cocktail studies address interactions between the major *T. reesei* cellulases [21, 61, 168, 355], some have also looked at thermostable CBHs and EGs from alternative fungal species, such as *M. thermophila*, *T. aurantiacus* and *Chaetomium thermophilum* [87, 128, 168]. In processes run at higher temperatures, higher conversion yields can be achieved with (optimized mixtures of) thermostable enzymes as compared with *T. reesei* enzymes [168]. LPMOs from thermophilic fungi, such as *Ta*AA9A from *T. aurantiacus* [134, 146, 148, 272, 284] and AA9 LPMOs from *M. thermophila* [114, 117], have gained considerable interest recently. *Ta*AA9A, for example, is a good candidate for being added to cellulase cocktails [250]; however, there is no publicly available information on whether it has been incorporated into today’s state-of-the-art commercial cellulase mixtures. Although thermostable enzymes have clear advantages in industrial settings, currently, no thermostable cellulase cocktails are available commercially [262].

#### Spiking studies to highlight enzyme activities lacking in commercial cellulase mixtures

Another, more direct approach to identify underperforming enzyme activities in cellulase cocktails is the supplementation or partial replacement of enzyme cocktails with either individual enzymes [134] or fungal broths [299]. An early example includes the supplementation of the *T. reesei*-derived Celluclast 1.5 cocktail with *A. oryzae*-produced Novozym 188 to compensate for the limited BG activity (e.g., in [299]). Analogously, several studies have shown co-operativity between commercially available cellulase, xylanase and pectinase preparations [19, 33, 119, 145], using combinations of products such as Accellerase 1000, Celluclast 1.5L, Spezyme CP, Multifect Xylanase, Multifect Pectinase and Viscozyme L. These studies add further proof to the general observation that no commercial cellulase preparation fits all substrates and highlight the importance of feedstock-specific enzyme blends.

To identify enzyme components that may be lacking in cellulase cocktails, commercial cellulase mixtures have also been supplemented with fungal culture broths or (semi)purified enzyme components. Celluclast has been studied extensively in spiking studies, revealing the positive impact of xylanase, mannanase and LPMO supplementation on the efficiency of cellulose saccharification [81, 87, 143, 177, 250, 272, 382], as also discussed above. In some cases, in-house fungal (e.g., *T. reesei*) culture broths have been used to showcase the positive effect of selected enzymes, such as three AA9s from *Geotrichum candidum* [205] or two AA14 LPMOs from *Pycnoporus coccineus* [68], on saccharification efficiency. The direct effects of these (purified monocomponent) enzymes will also have to be tested on the latest generation (hemi)cellulase cocktails for benchmarking.

The most recent commercial cellulase cocktails have also been subjected to spiking-type of studies. As an example, Agrawal et al. have shown that the performance of Cellic CTec2 on acid or alkali pretreated bagasse and rice straw can be boosted by addition of two AA9 LPMOs from the thermophilic fungi *Scytalidium thermophilum* and *Malbranchea cinnamomea* [8]. Very recently, von Freiesleben et al. have reported that supplementation with GH5 and GH26 mannanases leads to improved saccharification of pretreated lodgepole pine by Cellic CTec3 [367], confirming previous indications concerning suboptimal levels of mannanase activities in Cellic CTec3 for softwood saccharification [61]. As another example, d’Errico et al. showed that a Cellic CTec preparation and the β-glucanase preparation UltraFlo possess only low amounts of glucuronoyl esterase activity and that supplementing these products with CE15 glucuronoyl esterases boosts their saccharification efficiency on pretreated corn fiber [77]. The positive effect of CE15 supplementation on the saccharification yields varied with the substrate [77], further corroborating the importance of feedstock-specific enzyme blends.

### The interplay between process configuration and enzyme efficiency

The main considerations for process optimization entail (1) the type of feedstock and pretreatment method, (2) the choice of enzymes and their pH and temperature optima, (3) separate (SHF) or simultaneous (SSF) saccharification and fermentation steps, (4) stirring and aeration, (5) the possibility of on-site enzyme production and (6) possible measures for enzyme recycling. The choice of the process configuration (such as pretreatment, SHF/SSF and enzyme recycling) and physical parameters (such as temperature and level of dissolved oxygen) will have consequences for enzyme activity and stability. Of note, the enzymatic process is often separated into two phases: an initial liquefaction phase, in which the solid, particle-like feedstock becomes “fluid” (pumpable) and a saccharification phase, in which the polysaccharides are completely converted to soluble (mono-)sugars.

The choice of feedstock and pretreatment has a large impact on the type and amount of lignin remaining in the feedstock and, consequently, on the efficiency of both cellulases (in terms of the extent of unproductive binding) and LPMOs (in terms of delivery of reducing power). The temperature used during the enzymatic step(s) has to be carefully selected to compromise between enzyme efficiency and enzyme inactivation. Notably, the use of thermostable enzymes next to regular, less thermostable, cellulase cocktails will require alternative process configurations [363]. One possible scenario may be a liquefaction step run at elevated temperatures with a few selected thermostable enzymes, followed by full saccharification at lower temperature. In SSF, obviously, the temperature needs to be adapted to the fermenting microorganism. Of note, the impact of temperature goes beyond the impact on enzyme stability and activity, since temperature also affects potentially important abiotic factors such as reductant stability and dissolved oxygen levels, which may affect LPMO activity and/or the in situ generation of reactive oxygen species.

The improved efficiency of Cellic CTec2 compared to former, less efficient cellulase cocktails partly stems from the inclusion of LPMOs [146, 250]. The presence of molecular oxygen and/or H_2_O_2_ (Fig. 3) is crucial for LPMO activity, which will have to be considered in process design in general, and when choosing between SHF and SSF in particular. In a study comparing lactic acid production in different process setups, it was found that SHF performed better than SSF, and this was ascribed to the consumption of oxygen by the fermenting organisms in SSF, which lowered LPMO activity [249]. This is opposite to what has been observed in experiments with non‐LPMO-containing cellulase cocktails, where SSF processes tend to be more efficient [49, 230, 256, 344]. Interestingly, Cannella and Jørgensen showed that the relative performance of SSF and SHF approaches varied with substrate loading [49]. At 20% (w/w) substrate loading of wheat straw, SSF with LPMO-containing Cellic CTec2 performed better, but at 30% (w/w) substrate loading the SHF approach yielded more ethanol, possibly because LPMO activity, which is only expected in the SHF approach, becomes more important at higher substrate concentrations [49]. With the possibility of direct supply of low (i.e., non-lethal) amounts of H_2_O_2_ to saccharification reactions, in particular for low-lignin feedstocks, a more efficient SSF setup that fully harnesses the power of LPMOs may become possible, since this would avoid competition for oxygen between the fermenting organism and in situ generation of H_2_O_2_. However, so far no studies have been published on this topic.

Overall process economics and efficiency may be increased further by producing enzymes on site, instead of using (combinations of) commercially available cellulase cocktails [161]. The carbon source used in growth media has been shown to have clear impacts on the protein expression profile of fungal expression strains [255]. Thus, on-site enzyme production may allow for tailoring the cellulase cocktail (i.e., the composition of the fungal secretome) to the feedstock of the biorefinery, by using this feedstock as the carbon source when cultivating the cellulase expression strain [1, 255].

Since enzymes are catalysts and, in principle, could be used many times, enzyme recycling may be considered during process design [147, 164]. Enzyme recycling is a complex process that requires in-depth knowledge of enzyme–substrate interactions [346] and the mechanisms of enzyme adsorption–desorption [258, 280, 342, 358]. In principle, enzyme recycling could be done in two ways, either recycling the unhydrolyzed solid residue with bound enzymes or recycling the liquid phase with free (non-bound) enzymes [294, 295]. Both approaches have shown potential for saving enzyme costs [137, 293, 348], but they also make the process more complex. It is important to note that while enzyme recycling may seem attractive and “simple”, such recycling has some intrinsic limitations. At the end of the hydrolysis, key enzyme components may be diluted out in the recycled enzyme fraction as different enzyme components will remain free or adsorbed on the feedstock as well as become inactive to various degrees [215, 280, 358]. LPMOs likely suffer from autocatalytic inactivation, especially when substrate concentrations become low in the later phase of a degradation reaction (see above), whereas it is well known that certain cellulases may get “stuck” by non-productive binding to cellulose in an essentially irreversible fashion [156, 232, 259, 267].

Importantly, one of the current targets when optimizing saccharification setups concerns how to leverage LPMO activity while keeping LPMOs from inactivation. As discussed above, LPMO inactivation may be caused by reactive oxygen species that derive from reactions between O_2_ and lignin [185] or that are formed by the LPMO itself [37] or by other redox enzymes present in the enzyme mixture [39]. It has been shown for various reaction setups that too high feeding rates of externally added H_2_O_2_ [200, 248] or too high levels of in situ production of H_2_O_2_ [185, 269] lead to LPMO inactivation. Recent studies following the accumulation of LPMO products over the course of H_2_O_2_-assisted saccharification of industrial feedstocks [37, 65, 167, 248] clearly indicate that LPMO inactivation occurs presumably due to the accumulation of H_2_O_2_ in the reaction mixture, although the extent and rate of inactivation over time remain to be elucidated. Notably, there is a clear difference between LPMOs in terms of redox stability [66, 272], partly due to the presence or absence of CBMs (discussed below). Consequently, process robustness may be increased by screening for LPMOs with higher stability. Successful process optimization may further include control of the rate of addition or in situ generation of H_2_O_2_, control of dissolved oxygen levels, supplementation with catalase and/or superoxide dismutase to maintain low levels of H_2_O_2_ and superoxide radicals [37] as well as online monitoring and control of the redox processes taking place during saccharification, e.g., through online monitoring of the oxidation–reduction potential [167]. Before the power of LPMOs can be leveraged to its fullest extent, however, further fundamental research is required to better understand the impact of reactive oxygen species generated in biotic and abiotic redox processes on LPMO activity and to unravel the mechanisms of LPMO inactivation in the presence of industrially relevant feedstocks.

### The role of CBMs: for cellulases, hemicellulases and LPMOs

Many of the enzymes discussed above contain one, or sometimes more than one, additional domain referred to as carbohydrate-binding module (CBM) [40]. Such modules may bind to various faces of cellulose crystals, to the more amorphous regions of cellulose or to one or more hemicellulose types [51, 234]. Accordingly, some CBMs target surfaces (i.e., multiple polysaccharide chains, such as the CBM1 of *Tr*Cel7A), others target single polysaccharide chains, whereas the third type directs the catalytic domain to act at polysaccharide chain ends [123]. Substrate-binding by CBMs, while being fully reversible [90, 216, 267], may be very strong, because of which it has sometimes even been considered almost irreversible [52, 292]. Irreversible binding would be puzzling since it does not seem favorable for enzyme efficiency. There have been many theories about what CBMs do and how they work, including proposals that some CBMs may increase substrate accessibility by disrupting the crystalline structure of cellulose [40, 127]. The primary role of CBMs, with massive experimental support, is that they promote proximity between the appended catalytic domain and the substrate, thus promoting enzyme efficiency.

To some extent, CBMs and substrate binding are a double-edged sword in saccharification efficiency. On the one hand, CBMs increase the enzyme’s affinity to its substrate [184], which promotes enzyme activity on insoluble cellulose [345, 353]. For processive CBHs, the CBM has been proposed to promote the feeding of the cellulose chain into the CBH active site [184] and to increase processivity [25, 153, 181, 333], as well as to promote the stability of the CBH-cellulose complex. On the other hand, strong substrate binding via CBMs hinders desorption of bound enzymes [74, 333], which may get stuck on the substrate [199]. Moreover, CBMs contribute to unproductive binding of cellulases to lignin [286, 287, 321], which may result in enzyme inactivation.

The proximity effect of CBMs can be compensated by increasing substrate concentration, which will promote substrate binding of enzymes independent of the presence of a CBM. In 2013, Várnai et al. showed that, at high substrate concentrations, the truncated, CBM-free versions of the four CBM-containing cellulases from *T. reesei* (*Tr*Cel7A, 6A, 7B and 5A) were as efficient as the full-length enzymes [356]. Since then, the positive effect of increasing substrate concentration on the efficiency of cellulases and LPMOs without CBMs has been confirmed by a number of studies, as has the potentially negative impact of CBMs in reactions with high substrate concentrations [53, 66, 158, 170, 210, 334]. This observation can be explained by CBM-free cellulases having higher desorption rates (“off-rates”) [333] and reduced unproductive binding to lignin [260, 288], while increased substrate concentrations will overcome diffusional limitations of the CBM-free enzymes [372]. Of note, the presence or absence of CBMs in the enzyme components will affect potential enzyme recycling strategies. Using CBM-free enzymes will facilitate recycling unbound enzymes from the liquid phase [137, 258], while CBM-containing enzymes may be recycled in a bound form, with the unhydrolyzed solid residue and/or after desorption from the unhydrolyzed solid residue [211, 294, 295, 348].

CBMs also occur in LPMOs, although many LPMOs, including some of the best-studied ones with documented effects on cellulose saccharification [143, 178, 250, 284], lack CBMs. LPMO literature shows that certain single-domain LPMOs bind very well to their substrates, whereas recombinantly expressed catalytic domains of CBM-containing LPMOs sometimes seem to bind weakly [66, 110, 132]. It may thus seem that nature has evolved different strategies for LPMOs to have affinity for their substrates, but this is not yet sufficiently supported by systematic experimental studies. Existing data show that the CBMs of LPMOs have the same function as in GHs [53, 66, 72, 110, 111, 192, 209, 371] and it has also been shown that, like for GHs, the presence of a CBM becomes less important, and even unfavorable, when running reactions at high substrate concentrations [66].

Importantly, LPMOs that are reduced and meet O_2_ or H_2_O_2_ while not being bound to the substrate are prone to autocatalytic inactivation, due to the redox reactivity of the Cu(I) ion in the (reduced) catalytic center [37]. Thus, for LPMOs, proximity of the substrate not only promotes activity, but also stability, since proximity of the substrate increases the chances for the LPMO to engage in productive (i.e., oxidative cleavage of the substrate) rather than damaging side reactions. Several studies have shown that deletion of the CBM from a CBM-containing LPMO indeed leads to increased enzyme inactivation [66, 108, 273]. On the other hand, LPMOs have been found to bind more strongly to polysaccharides when the active site copper is in the reduced, i.e., Cu(I), state [188, 201], which is expected to favor their stability.

Interestingly, the importance of the proximity effect was also suggested by experiments with a cellulose-binding CBM-containing pyrroloquinoline quinone-dependent pyranose dehydrogenase (PDH) that can deliver reducing equivalents to LPMOs and thus drive the LPMO reaction. Upon removal of the CBM from this PDH, the LPMO reaction became less efficient and it has been suggested that this is due to proximity effects [357]. When the PDH is bound to cellulose, it will activate the LPMO while the LPMO is in close proximity to the substrate. On the other hand, a PDH that is free in solution will activate LPMOs that are not close to the substrate, thus increasing the chances for off-pathway reactions.

## Concluding remarks

Thanks to the efforts of a large research community and enzyme companies, today’s enzyme cocktails for saccharification of lignocellulosic biomass are so effective that industrial bioethanol production from such biomass has become a reality. Improved biomass pretreatment techniques have contributed to this development [391]. Despite much progress in the enzyme area, further improvements still seem possible. For example, it is still not fully clear how processive cellulases work and how the interplay of these essential but rather slow enzymes with other enzymes could be optimized [57, 169, 302, 306, 362]. Recent insights concerning the role of H_2_O_2_ and enzyme inactivation suggest that so far, we have not harnessed the full potential of LPMOs. Furthermore, despite much research on LPMOs in the past decade, exactly how these enzymes co-operate with classical cellulases remains largely unknown (see [343] for a recent study). Finally, recent work suggests that LPMOs could play a role in removing (traces of) recalcitrant hemicellulose, which may promote cellulolytic processes [68, 150]. On that note, further research on the impact of residual hemicellulose fractions in pretreated biomass and the possible roles of (any) hemicellulolytic enzymes in dealing with such fractions is still needed.

While research related to the enzymatic processing of lignocellulosic biomass has focused mainly on conversion of the polysaccharides, there is growing evidence that biomass saccharification and lignin modification by enzymes are interconnected [39]. Although our current understanding of enzymatic processing of lignin is still very limited, there is a growing interest in lignin valorization. As lignin constitutes nearly a third of plant biomass, the fate of the lignin fraction will need to be considered in the further development of biorefining processes for efficient and economic processing of lignocellulosic feedstocks [24, 285, 311]. A good example for the way forward is the so-called BALI process, where sulfite pretreatment generates both valuable carbohydrate and lignin streams which can be turned into valuable products [65, 301].

In addition to lignin valorization, there is a concerted ongoing research effort aimed at developing a widened portfolio of biomass-derived products, including cellulose-, hemicellulose-, and lignin-based polymers, oligomers and monomers, as well as products resulting from fermentation of lignocellulosic sugars, i.e., production of ethanol. Alternative fermentation products include microbial biomass for food and feed [29, 207, 336], alternative biofuels such as butanol [237] and commodity as well as high-value chemicals [317, 384]. In an environmentally and economically successful biorefinery, these products will co-exist as part of a flexible product portfolio that is continuously adjusted to feedstock availability, technological developments and market needs.
